# Immediate one-stage breast reconstruction for an 85-year-old breast cancer patient using deep inferior epigastric perforator flap surgery

**DOI:** 10.1093/jscr/rjab241

**Published:** 2021-07-10

**Authors:** Koshi Matsui, Toshihiko Satake, Misato Araki, Emi Kanaya, Takamichi Igarashi, Maki Okamoto, Takeshi Miwa, Katsuhisa Hirano, Toru Watanabe, Shinichi Sekine, Kazuto Shibuya, Isaya Hashimoto, Shozo Hojo, Isaku Yoshioka, Tomoyuki Okumura, Tsutomu Fujii

**Affiliations:** Department of Surgery and Science, Faculty of Medicine, Academic Assembly, University of Toyama, Toyama 9300194, Japan; Department of Plastic, Reconstructive and Aesthetic Surgery, University of Toyama, Toyama 9300194, Japan; Department of Surgery and Science, Faculty of Medicine, Academic Assembly, University of Toyama, Toyama 9300194, Japan; Department of Surgery and Science, Faculty of Medicine, Academic Assembly, University of Toyama, Toyama 9300194, Japan; Department of Surgery and Science, Faculty of Medicine, Academic Assembly, University of Toyama, Toyama 9300194, Japan; Department of Plastic, Reconstructive and Aesthetic Surgery, University of Toyama, Toyama 9300194, Japan; Department of Surgery and Science, Faculty of Medicine, Academic Assembly, University of Toyama, Toyama 9300194, Japan; Department of Surgery and Science, Faculty of Medicine, Academic Assembly, University of Toyama, Toyama 9300194, Japan; Department of Surgery and Science, Faculty of Medicine, Academic Assembly, University of Toyama, Toyama 9300194, Japan; Department of Surgery, Kamiichi General Hospital, Toyama 9300391, Japan; Department of Surgery and Science, Faculty of Medicine, Academic Assembly, University of Toyama, Toyama 9300194, Japan; Department of Surgery and Science, Faculty of Medicine, Academic Assembly, University of Toyama, Toyama 9300194, Japan; Department of Surgery and Science, Faculty of Medicine, Academic Assembly, University of Toyama, Toyama 9300194, Japan; Department of Surgery and Science, Faculty of Medicine, Academic Assembly, University of Toyama, Toyama 9300194, Japan; Department of Surgery and Science, Faculty of Medicine, Academic Assembly, University of Toyama, Toyama 9300194, Japan; Department of Surgery and Science, Faculty of Medicine, Academic Assembly, University of Toyama, Toyama 9300194, Japan

## Abstract

The deep inferior epigastric perforator (DIEP) flap is widely recognized as safe for use as a first-choice option in autologous tissue breast reconstruction; however, DIEP is often not performed for breast reconstruction in the elderly. We report a case of an 85-year-old woman who underwent DIEP flap reconstruction. Immediate reconstruction was performed after mastectomy. The patient successfully underwent DIEP flap reconstruction with no complications. Other options for reconstruction include a latissimus dorsi flap, a transverse rectus abdominis flap and implant-based reconstruction. DIEP flap reconstruction was performed, which does not cause muscle damage and provides sufficient volume. To our knowledge, this study is the first to report DIEP breast reconstruction in a patient over 85 years of age. This case demonstrates the usefulness of DIEP flap reconstruction for elderly patients.

## INTRODUCTION

Postmastectomy breast reconstruction is an important component of the comprehensive management of breast cancer patients. A large number of studies have demonstrated a significant increase in self-esteem, body image and quality of life after breast reconstruction in both young and older breast cancer survivors [1, 2]. From the American College of Surgeons, National Surgical Quality Improvement Program database, only 1.6% of patients over the age of 80 undergo breast reconstruction [3]. Age, tumor burden, Eastern Cooperative Oncology Group (ECOG) performance status and planned radiotherapy are the causes of not offering reconstruction. In particular, the likelihood of an offer decreases sharply once a woman is aged 70 years or more [4]. The National Institute of Health and Clinical Excellence guidelines state that breast reconstruction should be offered to all patients unless it is contraindicated. Here, we report an 85-year-old woman who safely underwent immediate reconstruction using a deep inferior epigastric perforator (DIEP) flap.

## CASE REPORT

**Figure 1:**
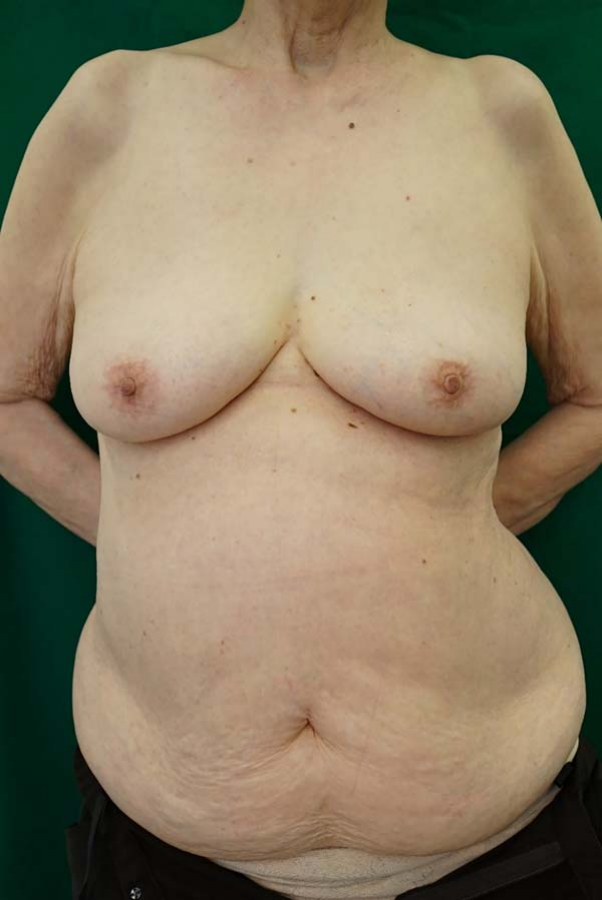
Before the operation.

**Figure 2:**
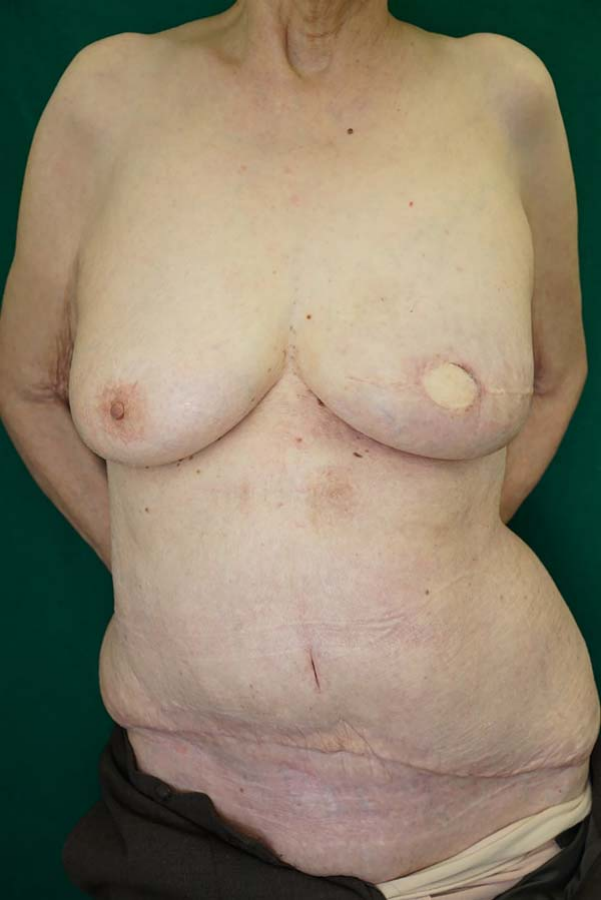
Three months after surgery.

An 85-year-old woman with a history of hypertension, hyperlipidemia and osteoporosis was diagnosed with ductal carcinoma of the right breast (Fig. 1). Her ECOG performance status was 1, and her body mass index was 26 kg/m^2^. Her main desire was immediate reconstruction with good esthetics and function without breast implants. Mastectomy followed by immediate breast reconstruction using a DIEP flap was planned. The weight of the specimen was 261 g. After tumor resection, a 12.0 × 49 cm free DIEP flap was elevated with a right side perforator. Indocyanine green angiography showed that the right perforator was enough to allow perfusion from zones I to III. The deep inferior epigastric artery and vein were anastomosed to the left internal mammary artery and vein at the third intercostal space. A flap inset was performed with a 180-degree clockwise rotation. The thick inset was trimmed twice. The monitoring flap was set to 4 cm, and the flap was fixed to the pectoralis major muscle. The operation time was 6 h and 2 min, and the amount of blood loss was 160 ml. The postoperative course was good, and the DIEP flap survived completely. No disease recurrence or discomfort was observed during 5 months of postoperative follow-up (Fig. 2).

## DISCUSSION

Breast reconstruction rates decrease significantly with increased age. One reason for the decline in breast reconstruction in older women may lie in surgeon perceptions of age and, consequently, a reluctance to discuss this option with patients. In addition, many patients do not wish to have breast reconstruction simply because of their age; however, age should not be a factor in whether to perform reconstruction as age alone is not associated with an increased operative risk [5]. Actual and physiological age may be vastly different, and other parameters should be used to better determine surgical safety. As patients are living longer after breast cancer treatment, a stronger emphasis has been placed on improving their quality of life [6].

Immediate breast reconstruction at the time of mastectomy has taken on greater popularity and is increasingly considered a standard of care [7]. Compared with a delayed approach, immediate breast reconstruction offers the potential benefit of fewer operations, decreased costs, better esthetic outcomes and reduced psychological distress for the patient [8]. Considering the surgical time, and technique, autologous reconstruction cannot be performed easily. Implant reconstruction is generally less invasive and can be performed with a shorter hospital stay; however, two surgeries are required to replace the expander with an implant, and there is a risk of breast implant-associated anaplastic large cell lymphoma in the long term. We aimed for a soft and natural breast, and we chose DIEP reconstruction in consideration of the patient’s wishes and general condition. Risk factors for complications of autologous reconstruction have been reported to be a higher American Society of Anesthesiologists classification, obesity and longer operation times [3]. The operation was performed with the same postoperative management as for younger patients aimed at an early hospital discharge. In fact, the patient was able to be discharged 7 days after surgery without complications.

Several complications have been reported after breast reconstruction. Bowman and colleagues retrospectively surveyed 61 older women who had undergone breast reconstruction under two named surgeons (latissimus dorsi [*n* = 8], pedicled TRAM [*n* = 43] and implant-based reconstruction [*n* = 31]), using the Short Form (SF-12) survey for physical and mental health. In the group that took 4 weeks to recover (39%), the majority (58%) had implant reconstruction. Of those who took between 4 and 8 weeks to recover (44%), 85% had autologous reconstruction. Only 8% of women took longer than 12 weeks to return to normal daily activities, and 100% of these patients had autologous reconstruction [9]. Mioton and colleagues reported that autologous reconstruction patients had more complications. Complications occurred in 4.4% of implant reconstructions and 8.7% of autologous reconstructions. Patients receiving autologous reconstruction were more likely to have a surgical complication, suffer a wound infection, experience flap failure, have wound disruption and undergo a reoperation [10]; however, in the long term, the rate of complications of autologous reconstruction decrease, and 5 years after the operation, reoperations increase with implant reconstruction [11]. Girotto *et al*. [6] note that autogenous tissue was preferable to implants in terms of pain and role limitation. A multicenter, retrospective analysis by Song *et al*. [12] assessing outcomes of autologous reconstructions in 1809 patients concluded that complications in elderly patients were ‘equivalent’ to those in younger women. Kamali and colleagues reported that the rates of breast reconstruction decrease with increasing age. Despite increasing age, the associated complication rates in immediate breast reconstruction patients remained stable [3]. According to Breast Q, the level of satisfaction with autologous reconstruction was higher than that with implant reconstruction [13].

## CONCLUSIONS

Breast reconstruction surgery should not be avoided simply because of advanced age. Various factors may influence the decision to offer or deny an elderly patient a particular procedure. More studies and longer follow-up periods will be required in the future, but the case presented here demonstrates the usefulness of DIEP flap reconstruction for elderly patients.

## CONFLICT OF INTEREST STATEMENT

None declared.

## FUNDING

None.

## References

[1] Butz DR, Lapin B, Yao K, Wang E, Song DH, Johnson D, et al. Advanced age is a predictor of 30-day complications after autologous but not implant-based postmastectomy breast reconstruction. Plast Reconstr Surg 2015;135:253e–61e. 10.1097/PRS.0000000000000988 25626808

[2] Lee C, Sunu C, Pignone M. Patient-reported outcomes of breast reconstruction after mastectomy: a systematic review. J Am Coll Surg 2009;209:123–33. 19651073 10.1016/j.jamcollsurg.2009.02.061 PMC2721826

[3] Kamali P, Curiel D, van Veldhuisen CL, Bucknor AEM, Lee BT, Rakhorst HA, et al. Trends in immediate breast reconstruction and early complication rates among older women: a big data analysis. J Surg Oncol 2017 Jun;115:870–7. 28409847 10.1002/jso.24595

[4] Jeevan R, Browne JP, Gulliver-Clarke C, Pereira J, Caddy CM, van der Meulen JH, et al. Association between age and access to immediate breast reconstruction in women undergoing mastectomy for breast cancer. Br J Surg 2017;104:555–61. 28176303 10.1002/bjs.10453

[5] Audisio RA, Corsini G, Gennari R, Hoekstra HJ, Maffezzini M, Mobarak D, et al. Pre-operative assessment of cancer in the elderly (PACE): a comprehensive assessment of underlying characteristics of elderly cancer patients prior to elective surgery. Surg Oncol 2007;15:189–97. 10.1016/j.suronc.2007.04.009 17531743

[6] Girotto JA, Nahabedian MY, Schreiber J. Breast reconstruction in the elderly: preserving an excellent quality of life. Ann Plast Surg 2003;50:572–8. 12783001 10.1097/01.SAP.0000069064.68579.19

[7] Butler PD, Nelson JA, Fischer JP, Wink JD, Chang B, Fosnot J, et al. Racial and age disparities persist in immediate breast reconstruction: an updated analysis of 48,564 patients from the 2005 to 2011. Am J Surg 2016;212:96–101 2014; 21:3290–6. 26545345 10.1016/j.amjsurg.2015.08.025

[8] Sullivan SR, Fletcher DR, Isom CD, Isik FF. True incidence of all complications following immediate and delayed breast reconstruction. Plast Reconstr Surg 2008;122:19–28. 18594356 10.1097/PRS.0b013e3181774267

[9] Bowman CC, Lennox PA, Clugston PA, Courtemanche DJ. Breast reconstruction in older women: should age be an exclusion criterion? Plast Reconstr Surg 2006;118:16–22. 16816669 10.1097/01.prs.0000220473.94654.a4

[10] Mioton LM, Smetona JT, Hanwright PJ, Seth AK, Wang E, Bilimoria KY, et al. Comparing thirty-day outcomes in prosthetic and autologous breast reconstruction: a multivariate analysis of 13,082 patients? Plast Reconstr Aesthet Surg 2013;66:917–25. 10.1016/j.bjps.2013.03.009 23562485

[11] Rusby JE, Waters RA, Nightingale PG, England DW. Immediate breast reconstruction after mastectomy: what are the long term prospects? Ann R Coll Surg Engl 2010;92:193–7. 20223055 10.1308/003588410X12628812458770 PMC3080066

[12] Song D, Slater K, Papsdorf M, Van Laeken N, Zhong T, Hazen A, et al. Autologous breast reconstruction in women older than 65 years versus women younger than 65 years. Ann Plast Surg 2015;76:155–63. 10.1097/SAP.0000000000000527 26637165

[13] Toyserkani NM, Jorgensen MG, Tabatabaeifar S, Damsgaard T, Sorensen JA. Autologous versus implant-based breast reconstruction: a systematic review and meta-analysis of Breast-Q patient-reported outcomes. J Plast Reconstr Aesthet Surg 2020;73:278–85. 31711862 10.1016/j.bjps.2019.09.040

